# Biologic and genomic characterization of a novel virulent *Aeromonas hydrophila* phage phiA051, with high homology to prophages

**DOI:** 10.3389/fvets.2024.1415685

**Published:** 2024-07-18

**Authors:** Yuzhi Wang, Guixiang Tong, Xinglong Jiang, Chuandeng Tu, Hongjiao Cai, Wenhong Fang, Honglian Tan, Qibiao Weng, Xinxian Wei, Mao Lin

**Affiliations:** ^1^State Key Laboratory of Mariculture Breeding, Fisheries College of Jimei University, Xiamen, China; ^2^Guangxi Key Laboratory of Aquatic Genetic Breeding and Healthy Aquaculture, Guangxi Academy of Fishery Sciences, Nanning, China; ^3^Engineering Research Center of the Modern Technology for Eel Industry, Ministry of Education, Xiamen, China; ^4^Xiamen Key Laboratory of Intelligent Fishery, Xiamen Ocean Vocational College, Xiamen, China; ^5^East China Sea Fisheries Research Institute, Chinese Academy of Fisheries Sciences, Shanghai, China; ^6^Key Laboratory of Eel Aquaculture and Processing of Fujian Province, Fuzhou, China

**Keywords:** *Aeromonas hydrophila*, bacteriophage, biological characteristics, whole genome, prophage

## Abstract

**Introduction:**

*Aeromonas hydrophila* is particularly harmful to freshwater aquaculture, and the search for phage is an effective biological control method, but reports of possible temperate phages and their mutants are rare in this field. In this study, a virulent phage highly homologous to prophage in the genomes of *A. hydrophila* was collected and preliminary biological characterization was carried out to understand its nature.

**Materials and methods:**

Water samples taken from eel ponds in Fujian, China were combined with the strain. Spot test method and double-layer agar plate assay was used for confirmation and purification. Phage virions were observed using transmission electron microscope. A total of 68 strains of *Aeromonas spp*. were used to determine the host range. MOI groups of 1,000, 100, 10, 1, 0.1, 0.01, 0.001, 0.0001, 0.00001 were prepared to detect the optimal MOI. The conditions of thermal stability assay were set as 30, 40, 50, 60, 70 and 80°C for 1 h, respectively, and conditions of acid and alkali stability assay were set as 2.0, 4.0, 6.0, 8.0, 10.0 and 12.0 of pH. MOI of 0.01 and 0.1, respectively, are set to determine the inhibitory capacity of phage.

**Results:**

A novel virulent *A. hydrophila* phage designated phiA051 has been isolated from aquaculture water. Electron microscopic observation showed that the phage phiA051 was composed of an icosahedral capsid. The phage phiA051 possesses an optimal multiplicity of infection (MOI) of 0.01, and its burst size was 108 PFU/cell. The phage maintained a high viability at temperatures of 30–50°C or pH 6.0–10.0 for 1  h. Phage phiA051 has certain potentials in rapidly inhibiting the spread of pathogen early in the outbreak, and it has a linear dsDNA with GC content of 60.55% and a total length of 32,212  bp, including 46 ORFs.

**Discussion:**

The phage phiA051 behaved as a virulent phage. However, the BLASTN result showed that 23 of the top 25 hits were genomes of Aeromonas strains. It was suggested that phiA051 was probably derived from some prophage in the chromosome of Aeromonas. Further investigation of the mechanism how phage phiA051 transforms from a temperate phage to a virulent phage will provide a unique perspective and idea to explore the potential of prophages.

## 1 Introduction

Diseases caused by bacterial infections are one of the major threats to the health of animals and human beings (1, 2). Antibiotics have been extensively used to control bacterial infections for a long time. However, the emergence of antimicrobial-resistant bacteria has greatly reduced their effectiveness and led to numerous deaths. Therefore, researchers have been committed to finding alternative therapies to antibiotics (3, 4). One potential option is the use of phages, which are viruses that exclusively infect bacteria (5). Phages are categorized into virulent and temperate phages based on the difference in their lysis cycle. Virulent phages do not enter a stage of integration with the host bacterial genome, while temperate phages integrate their genome into the host bacterial chromosome and may re-enter the lytic cycle under certain conditions. The integrated DNA is referred to as a prophage. Temperate phages are difficult to apply clinically as they are unable to kill bacteria stably, and the integration of them can lead to horizontal gene transfer, including transfer of antibiotic resistance genes or virulence factors. Such gene transfer could inadvertently increase pathogenicity or resistance of bacterial population, so only virulent phages are prioritized for screening and consideration for phage therapy (6–12).

*Aeromonas hydrophila*, a Gram-negative facultative anaerobic bacterium, is a common pathogen of freshwater farmed animals (13–16). It is pathogenic to a wide range of fish, amphibians and reptiles and can cause systemic and ulcerative infections, including septicemia, gill rot and kidney disease (17–19). In addition, it can infect terrestrial animals and even humans (20–22). Given the effectiveness of phage therapy against bacteria (8, 10, 12, 23, 24), some researchers have also carried out studies on *Aeromonas* phages, mainly focusing on the elaboration of phage screening and bactericidal capabilities, for example the host ranges of several virulent *Aeromonas* sp. phages were delineated (25–27), and methodologies for the augmentation of aquatic phages have also been investigated by researchers (9, 28–32). Despite a number of studies, the amount of phage species that have been discovered and studied remain relatively small. There are currently 322 genomes of *A. hydrophila* strains in the National Center for Biotechnology Information (NCBI) database, whereas there are only 300 genome records for phages targeting this bacterium, and even fewer phages with documented taxonomic status. Phages of *A. hydrophila* are now known to belong mostly to the families *Autographiviridae*, *Chaseviridae*, *Demerecviridae*, *Ackermannviridae*, *Straboviridae*, *Casjensviridae* and *Peduoviridae*. The continued exploration of phage bioresources is necessary due to the limitations of many known phage species for applications such as rather narrow lysis spectrum, low lysis volumes and short inhibition times (8, 12, 33–35). Since it has been shown in recent years that some virulent phages can be obtained by gene editing of temperate phages (36, 37), it has become very important to study the mechanism of natural and artificial transitions from temperate to virulent phages, which can not only help people to gain a deeper understanding of the survival mode and properties of phages, but also help them to obtain potentially virulent phages as much as possible by means of mutation.

In this study, a virulent phage with high homology to a series of prophages was obtained during the screening of virulent phage targeting *A. hydrophila*. This phage is not capable of lysogeny switching, and from this it should be considered as a virulent phage. However, during the later genome sequence comparison we found that this phage has a high degree of homology with many *Aeromonas* prophage genomes, suggesting that this phage may originate from a temperate phage, but the mechanism of this transition is not clear. In the future it can be an important material to study the evolution of temperate phage into virulent phage.

## 2 Materials and methods

### 2.1 Isolation and purification

Water samples were taken from eel ponds in Fujian, China, aiming to detect phages against *A. hydrophila* A051, a strain isolated from diseased eels (38). The host A051 was inoculated into 100 mL of LB broth and incubated at 30°C, 150 rpm for 12 h to logarithmic phase, at which time the concentration of the bacterial suspension was approximately 10^9^ CFU/mL. Water samples (1 L) were combined with 50 mL culture of the strain A051 and 800 mL of Luria–Bertani (LB) broth, then the mixture was cultured for 24 h at 30°C with intermittent stirring to enrich possible phages. Following this, 10 mL of the mixture was centrifuged for 5 min at 4°C, 10,000 rpm. The supernatant was filtered with a 0.22 μm filter membrane, and spot test method was applied to seek out phages according to the method from Zhang et al. (39). Once a phage plaque had formed, a double-layer agar plate assay was used for confirmation and purification of the phage according to the method from Ye et al. (38). Purification procedure was repeated 5 times. Purified phage was stored at 4°C.

### 2.2 Virion morphology

The purified phage stock (approximately 1.0 × 10^9^ PFU/mL) was placed on paraffin film with a copper mesh and left for 30 min. The film was negatively stained with 20 μL 1% (w/w) phosphotungstic acid solution for 3 min subsequently. Excess filtrate and staining solution were then removed. Phage virions were observed using transmission electron microscope (JEOL Co., Tokyo, Japan) and the head length, head width and tail length were measured.

### 2.3 Host range

A total of 68 strains of *Aeromonas* spp. respectively designated as A001 ~ A068 were used to determine the host range, which included 24 strains of *A. hydrophila*, 20 strains of *A. veronii* and 24 strains of other *Aeromonas* spp. Spot test was used to verify if the strain could be a host. Both strains source and test method were identical to those used by Ye et al. (38). The formation of phage plaques confirmed the strain as a host.

### 2.4 Multiplicity of infection

The bacterial suspension was estimated by OD_600_ value based on the OD-concentration standard curve in the pretest, and the titer of stock phage was determined using spot test. Host bacterial dilutions and phage dilutions for MOI groups of 1,000, 100, 10, 1, 0.1, 0.01, 0.001, 0.0001, 0.00001 were prepared according to Liu et al. (23). Thereafter, 100 μL of both bacterial and phage dilutions of each group were taken, respectively, and mixed with 800 μL of LB broth. The mixture was incubated for 5 h at 30°C, 180 rpm. The double-layer agar assay was employed to calculate the phage titer. The MOI giving the highest final phage titer was deemed the optimal MOI (OMOI). All groups were performed in triplicate.

### 2.5 One-step growth curve

Following the method from Zhang et al. with some modifications, host A051 was cultured to the logarithmic phase, then 5 mL of phage suspension and 5 mL of A051 was then mixed with an equal volume of it at the optimal MOI of 0.01, incubated at 30°C for 10 min. The bacteria-phage mixture was centrifuged for 2 min at 10,000 rpm, then the supernatant was discarded and the sediment was resuspended with 10 mL LB broth and cultured at 30°C, 150 rpm. The 100 μL of culture was pipetted out at 10, 20, 30, 40, 60, 90, 120 and 150 min, respectively, and diluted in PBS prior to enumeration using the double-layer plate assay. The phage titer of each sample was determined in triplicate.

### 2.6 Stability to heat and acid/alkali

For thermal stability assay, 1 mL of phage suspension with a titer of 5 × 10^7^ PFU/mL was incubated at 30, 40, 50, 60, 70 or 80°C for 1 h, respectively. For acid and alkali stability assay, the solution with pH value of 2.0, 4.0, 6.0, 8.0, 10.0 or 12.0 were prepared using PBS buffer, 0.2 mol/L Na_2_HPO_4_·12H_2_O and 0.1 mol/L citric acid. 900 μL of each solution was mixed with 100 μL phage suspension with a titer of 5 × 10^7^ PFU/mL, respectively, and incubated for 1 h at 30°C. Phage titers of all samples were determined using double-layer plate assay and performed in triplicate.

### 2.7 Inhibitory capacity against *Aeromonas hydrophila* A051

*Aeromonas hydrophila* A051 was inoculated into 100 mL of LB broth and cultured until the OD_600_ value of the culture is 0.14 (approximately 1.2 × 10^8^ CFU/mL of bacterial density), then was mixed with phage phiA051 at a 1:1 volume ratio at a starting MOI of 0.01 and 0.1, respectively. 100 μL of mixture was transferred to each well of a 96-well plate with 100 μL of LB broth. The culture of bacteria alone with 100 μL of LB broth was used as a control group. The plate was incubated in a multi-plate reader at 30°C for 24 h with shaking, and the OD_600_ value of each well was measured at the interval of 30 min during incubation. Each treatment was performed in triplicate.

### 2.8 Genomic sequencing

Phage DNA was extracted following the directions provided by the TIANamp Virus DNA/RNA Kit (Tiangen Biotech Co., Ltd., Beijing) and was sequenced by Illumina Hiseq sequencing system at Majorbio (Shanghai, China). The bacterial genome scaffolds were assembled by splicing the optimized sequences after second-generation sequencing with multiple K-mer parameters using the short sequence assembly software SOAPdenovo2^1^ (41) to obtain the optimal contigs results, and then comparing the reads to the contigs, local assembly and optimization of the assembly results were carried out to form the scaffolds according to the paired-end and overlap relationships of the reads. The completed genome map was assembled by using the assembly software unicycler v0.4.8 (42) to assemble the three-generation sequences, and the sequences were corrected with the help of the software pilonjin. Coding sequences (CDS) in the genome were predicted using Glimmer (43).^2^ Prodigal. tRNAscan-SE v2.0^3^ (44) was used to predict tRNAs contained in the genome. ResFinder (45) was used to predict genes mediating antimicrobial resistance in phage DNA, and VFDB (46) was used to predict the presence of virulence factors.

### 2.9 Data analysis

The phage concentration data used for coordinate mapping were converted to common logarithms and plotted using GraphPad Prism 9.0.0 (121). Each coordinate point represented the mean value of triplicates, and the error bar indicated the standard deviation. GeneMarkS^4^ and EasyFig (47) were used for gene prediction and Co-linearity analysis of phage genome, respectively. The predicted genes were annotated using the online BLASTP tool (NCBI)^5^ and GeneMarkS (see text footnote 4, respectively). The whole genome circle map was graphed using Proksee.^6^ MEGA 11.0 (48) and VIPtree^7^ were used to construct and visualize the phylogenetic tree. PHASTER and Prophage Hunter were used to indicate prophage (49, 50).

## 3 Results

### 3.1 Morphology

**Figure 1:**
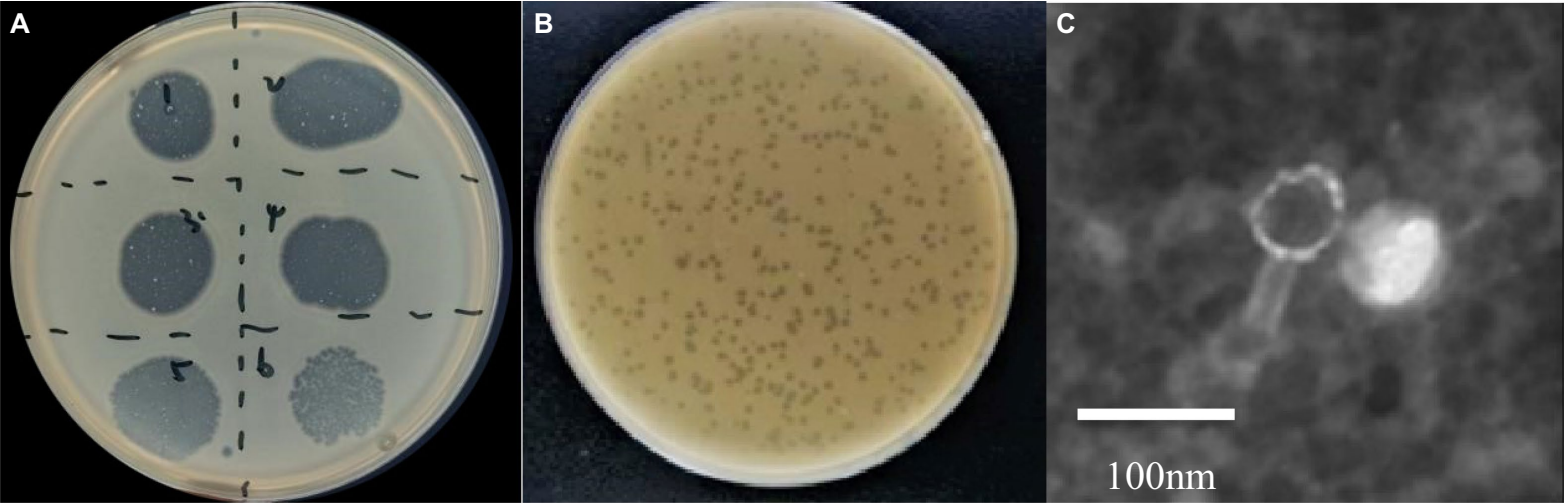
Morphology of phage phiA051. **(A)** Plaque formation by spot test, dilutions from number 1–6 are 10^−1^, 10^−2^, 10^−3^, 10^−4^, 10^−5^, 10^−6^, respectively. **(B)** The appearance of plaque on the double-layer agar. **(C)** The TEM graphs of negatively-stained. Bar, 100 nm.

A phage targeting *A. hydrophila* A051 was isolated and designated as phiA051. Without any inducer, the phage could be cultured continuously for more than 20 passages, with stable formation of phage plaques and no bacterial colonies in the phage plaques (Figure 1A). Diameter of the plaque was 0.32 ± 0.06 cm on the double layer plate (Figure 1B). The phage phiA051 was composed of an icosahedral capsid with an isometric dimension of 71.3 ± 2.0 nm, and a 73.1 ± 2.7 nm tail, imaged by transmission electron microscope (TEM) (Figure 1C).

### 3.2 Host range

Phage phiA051 could only lyse *A. hydrophila* A009, A013, A019 and A051 out of 68 tested *Aeromonas* strains (including 24 strains of *A. hydrophila*), implying that it has a narrow host range compare with some of phages that have been reported (38, 51–59).

### 3.3 Multiplicity of infection

The optimal MOI for phage phiA051 was 0.01, as indicated by the fact that phage phiA051 achieved the highest final titer (1.21 × 10^9^ PFU/mL) in this infected group (Table 1).

**Table 1:** Proliferation ability of phage phiA051 at different multiplicity of infection.

MOI	Initial concentration		Final concentration
	Phage (PFU/mL)	Host bacteria (CFU/mL)	Phage (PFU/mL)
1,000	2.0 × 10^8^	2.0 × 10^5^	7.70 × 10^7^
100	2.0 × 10^8^	2.0 × 10^6^	8.80 × 10^7^
10	2.0 × 10^8^	2.0 × 10^7^	1.14 × 10^8^
1	2.0 × 10^8^	2.0 × 10^8^	1.51 × 10^8^
0.1	2.0 × 10^7^	2.0 × 10^8^	1.61 × 10^8^
0.01	2.0 × 10^6^	2.0 × 10^8^	1.21 × 10^9^
0.001	2.0 × 10^5^	2.0 × 10^8^	2.14 × 10^8^
0.0001	2.0 × 10^4^	2.0 × 10^8^	2.00 × 10^8^
0.00001	2.0 × 10^3^	2.0 × 10^8^	1.98 × 10^8^

### 3.4 One-step growth curve

**Figure 2:**
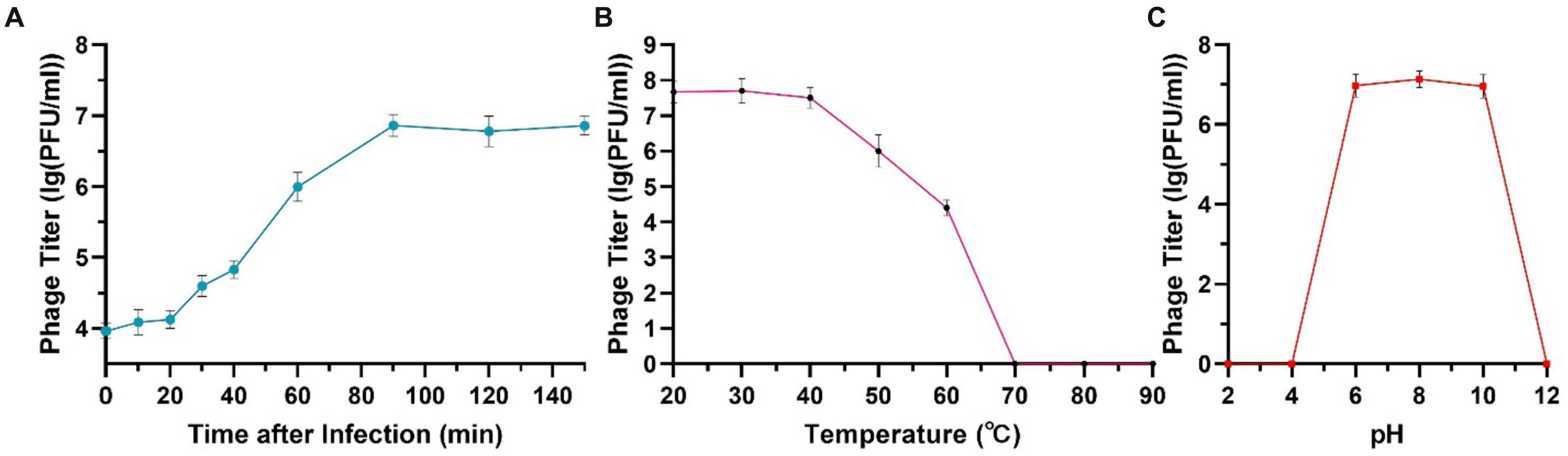
One-step growth curve **(A)**, thermal **(B)** and acid-alkali **(C)** stability of phage phiA051. Error bars represent the standard deviation of the mean.

The one-step growth curve of phage phiA051 indicates that the phage first undergoes a latent phase of 20 min after absorption with the host bacterium, followed by a lysis phase of 70 min, and finally enters a plateau phase at 90 min. The burst size of this phage was 108 PFU/cell, indicating that each infected host cell could produce 108 phage progenies on average (Figure 2A).

### 3.5 Stability to heat and acid/alkali

The phage had a high survival rate in the range of temperature between 30°C and 50°C. The viability decreased with the increase of temperature. When the temperature rose to 70°C, all phages were inactivated (Figure 2B). Phage phiA051 had an acid-alkali tolerance range of pH 6.0–10.0 (Figure 2C).

### 3.6 Inhibitory capacity against *Aeromonas hydrophila* A051

**Figure 3:**
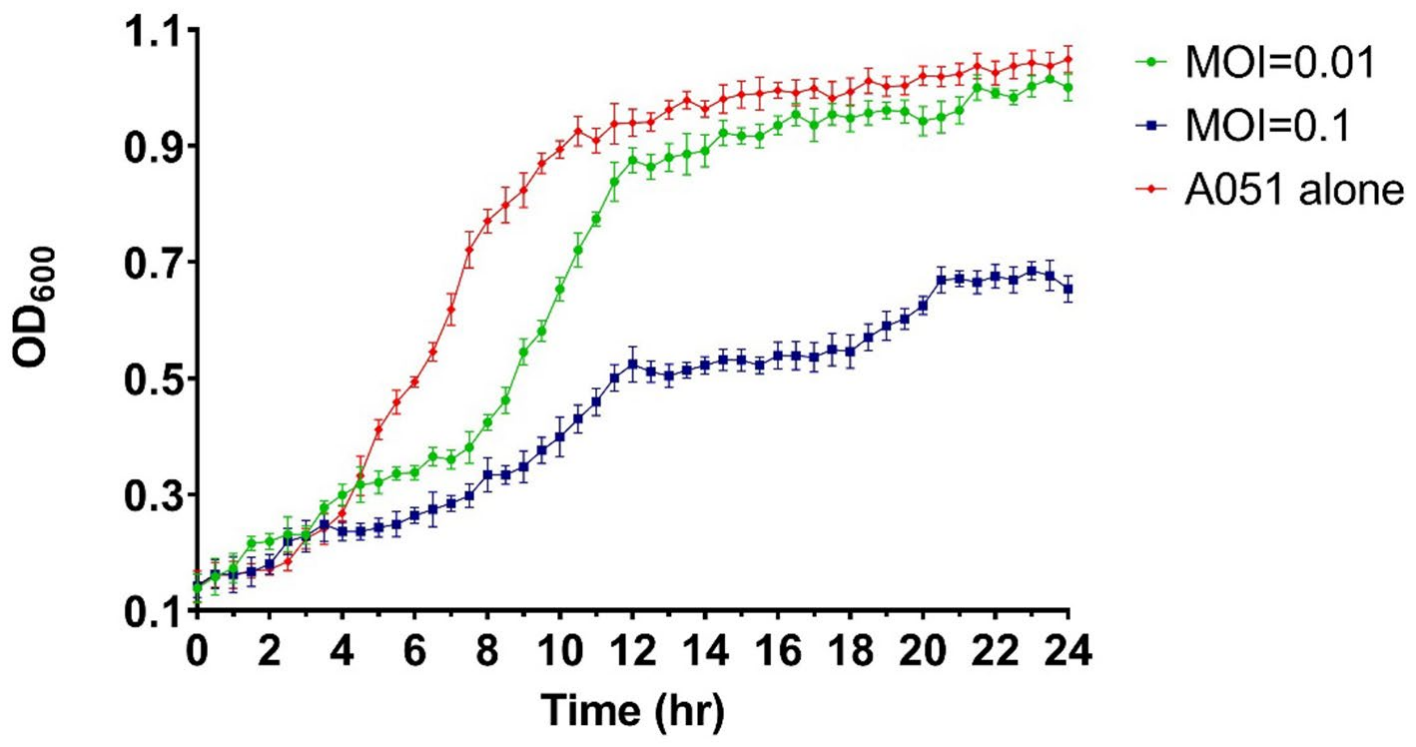
Bacteriostatic effect of phage phiA051 against *A. hydrophila* A051 at MOI of 0.1 and 0.01 *in vitro*. The concentration of bacteria was indirectly reflected by the OD_600_ value. Error bars represent the standard deviation of the mean.

Under the co-culture conditions of phage and *A. hydrophila* A051, the MOI = 0.01 group did not have significant inhibition and only showing limited suppression in the pre-logarithmic period of growth of A051, whereas the MOI = 0.1 group suppressed the growth of A051 throughout the whole process after 4 h. After 24 h of incubation, the OD_600_ of MOI = 0.1 group was only 62.4% of that of the control group. The result indicate that the amount of phage has an effect on the inhibition effect, and a larger amount of phage within a certain limit is more effective (Figure 3).

### 3.7 Genome outline and annotation

**Figure 4:**
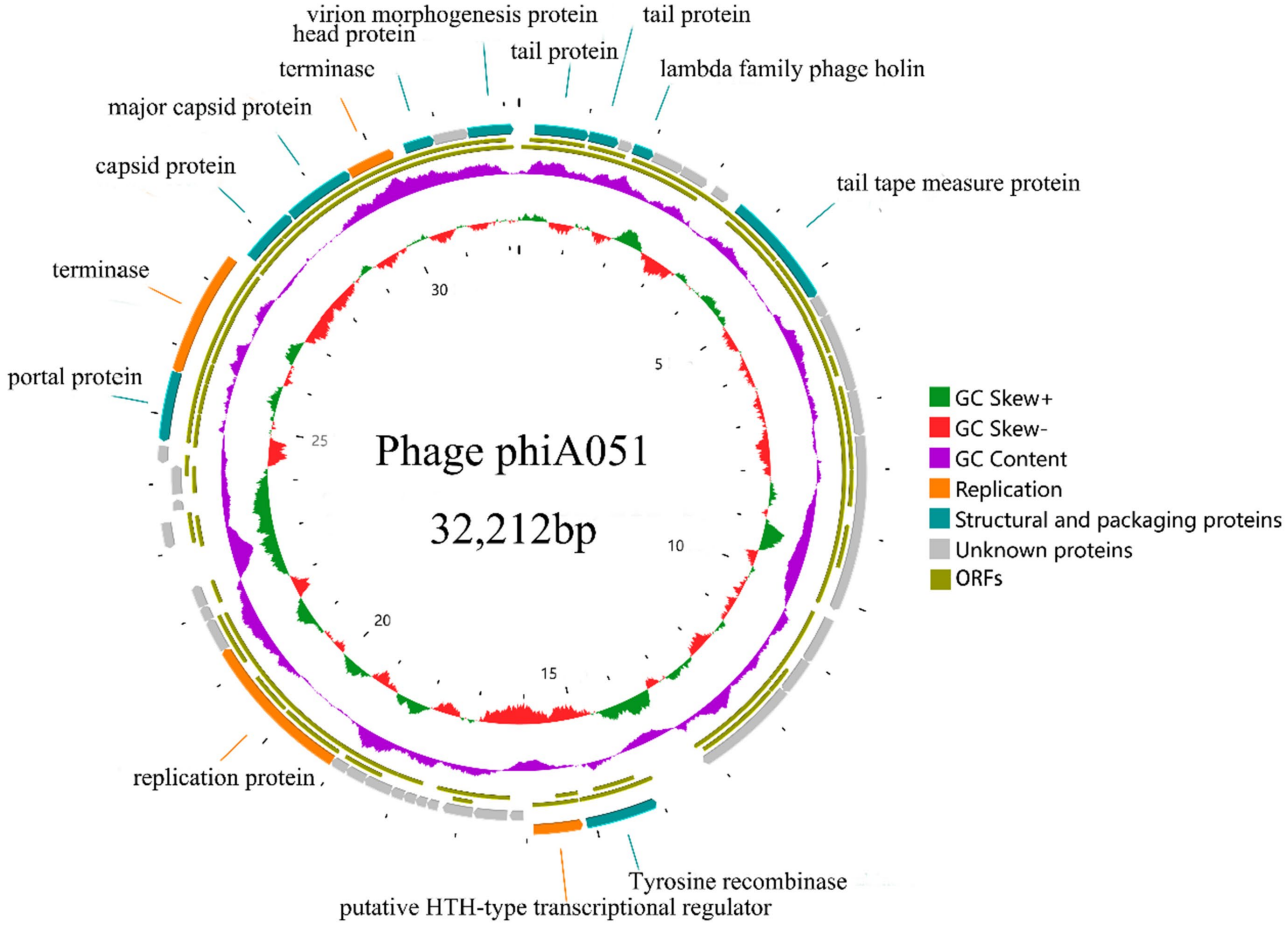
Circularized genomic map of phage phiA051. The number at the innermost circle denotes positions. The inward peaks of GC skew ± (known as G-C/G + C) indicating a greater proportion of G. The clockwise arrow with an inner position indicates the forward reading frame.

Next,-generation sequencing results show that the genome of phage phiA051 is a linear double-stranded DNA (dsDNA) comprising 32,212 bp and a GC content of 60.55% (Figure 4). There were 46 open reading frames (ORFs), including 26 functional ORFs assigned a function until now and 20 hypothetical proteins (Table 2). Among the functional ORFs, there are many important proteins for phage, such as the integrase (ORF19) which may play an important role in regulating the transition between the lysis and lysogenic cycles, the two terminase subunits (ORF39, 42) involving in DNA translocation and head-filling, Rz1-like lysis system protein LysC (ORF07) and holin (ORF04) associated with lytic infestation, TraR/DksA family protein (ORF03) and helix- turn- helix regulator (ORF20) related to the replication of DNA, tail protein (ORF01,14), portal protein (ORF38), capsid protein (ORF40, 41), head protein (ORF44) and virion morphogenesis protein (ORF46) as structure proteins. No putative toxins and antimicrobial resistance could be found.

**Table 2:** Annotation of open reading frames in the genome of phage phiA051 by BLASTP.

ORF	Size (bp)	Predictive function	Accession number	Coverage (%)	Identity (%)
ORF01	819	Tail protein	WP_039040908	100	98.5
ORF02	456	Tail protein	WP_039040909	100	100
ORF03	210	TraR/DksA family transcriptional regulator	WP_102949114	100	100
ORF04	321	Holin	KLV47711	99.06	97.1
ORF05	480	TIGR02594 family protein	WP_161648013	100	100
ORF06	432	Phage protein	KGY69175	100	95.1
ORF07	150	Rz1-like lysis system protein LysC	WP_323950247	100	87.8
ORF08	264	Phage tail assembly chaperone	WP_045529830	100	100
ORF09	144	Hypothetical protein	YP_001285660	100	87.2
ORF10	1773	Phage tail tape measure protein	WP_024943411	100	92.2
ORF11	324	DUF2590 family protein	WP_163147977	100	100
ORF12	1,188	Baseplate J/gp47 family protein	WP_201964397	100	99.0
ORF13	681	Tail protein	WP_241344190	100	94.7
ORF14	2,664	Phage tail fiber-like protein	AGM45696	41.49	81.5
ORF15	726	Hypothetical protein	WP_017782558	93.78	66.8
ORF16	561	Phage protein	WP_039040920	100	91.4
ORF17	1,611	Phage protein	WP_039040921	100	95
ORF18	225	Hypothetical protein	-	-	-
ORF19	1,053	Integrase	YP_001285625	100	98.9
ORF20	543	Helix-turn-helix transcriptional regulator	MBF8452010	100	99.4
ORF21	216	Phage protein	KGY69201	100	100
ORF22	510	Phage regulatory CII family protein	WP_161647994	100	99.4
ORF23	459	Hypothetical protein	WP_039040890	100	98.7
ORF24	177	Hypothetical protein	ALP41834	84.48	63.3
ORF25	174	Hypothetical protein	KGY73685	100	100
ORF26	192	Hypothetical protein	WP_039040892	100	98.4
ORF27	210	Hypothetical protein	KGY69196	100	100
ORF28	429	Hypothetical protein	WP_049636494	97.89	94.2
ORF29	315	Hypothetical protein	KGY69194	100	100
ORF30	258	Hypothetical protein	KGY69193	100	100
ORF32	504	Hypothetical protein	KGY69606	97.17	98.7
ORF33	225	Hypothetical protein	KGY69192	100	98.2
ORF34	333	Phage protein	KGY69191	100	100
ORF35	378	Helix-turn-helix transcriptional regulator	WP_201871554	96	99.17
ORF36	165	Hypothetical protein	KGY69189	96.8	98.3
ORF37	435	Hypothetical protein	WP_061182292	92.59	80
ORF38	1,014	Phage portal protein	KGY69188	100	99.3
ORF39	1815	Terminase large subunit	KGY69187	100	99.4
ORF40	864	Phage capsid protein	WP_039040901	100	99.5
ORF41	1,077	Major capsid protein	WP_039040902	100	95.5
ORF42	726	Terminase small subunit	WP_039040903	100	99.4
ORF43	96	Hypothetical protein	–	–	–
ORF44	462	Head protein	WP_039040905	100	100
ORF45	525	Tail protein	WP_039040906	97.7	98.8
ORF46	687	Phage virion morphogenesis protein	WP_039040907	100	99.1

### 3.8 Genome alignment and prophage analysis

**Figure 5:**
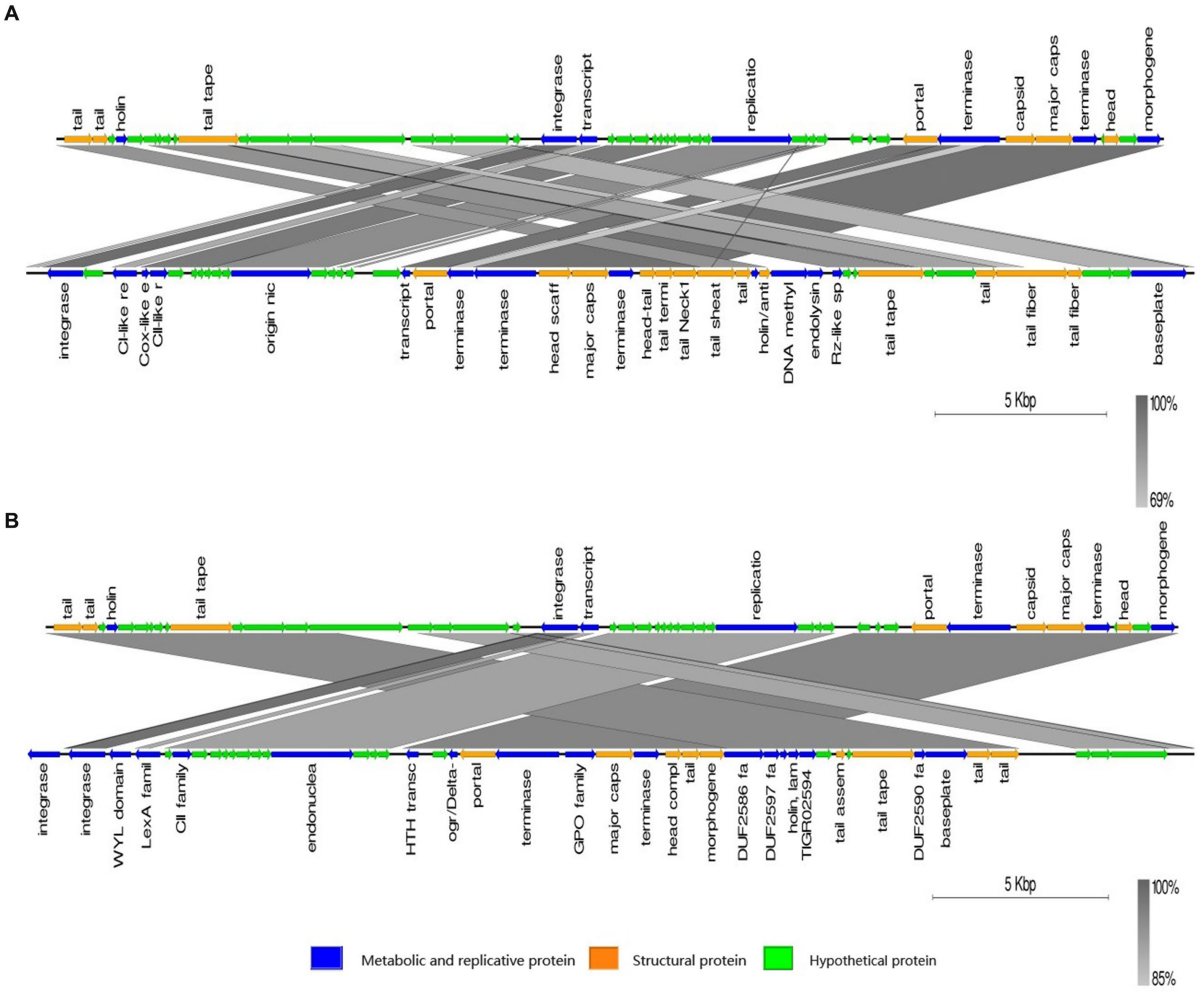
Visualized co-linearity analysis analysis between phage phiA051 (upper) and phage phiO18P **(A)**, phage phiA051 (upper) and prophage in *Aeromonas caviae* SS332 **(B)**. Right arrow indicates the forward reading frame.

The genome of phage phiA051 was analyzed using BLASTN and most of the query hits were prophages, displaying as bacterial genomic sequences of *Aeromonas* spp. Of the top 25 hits, there were 23 prophages in bacterial chromosomes and only 2 phage isolates (Table 3). The most similar nucleotide sequence was the prophage coming from the 3,003,688 - 3,033,875 bp fragment of the *Aeromonas caviae* SS332 genome, with an overall similarity of 84.9% (% identity multiplied by % coverage). And the closest phage isolate was *Aeromonas* phage phiO18P, with an overall similarity of 70.5%. The nucleotide sequences of them were selected for synthetic analysis with phiA051, respectively. Co-linearity analysis of phiA051 and phiO18P genomes showed two homologous fragments for phiA051 and phiO18P. Co-linearity analysis of phiA051 and SS332 showed that phiA051 and SS332 have three homologous fragments (Figure 5).

**Table 3:** Top 25 hits queried with the genome of phage phiA051 by BLASTN.

Description name	Coverage (%)	Identity (%)	Length (bp)	Accession number
*Aeromonas caviae* SS332	88	96.49	4,791,337	CP071151
*Aeromonas dhakensis* KN-Mc-6 U21	81	97.14	4,868,053	CP023141
*Aeromonas caviae* LZSFT54	90	94.89	4,675,189	CP133757
*Aeromonas caviae* WP5-W18-ESBL-02	85	96.01	4,751,792	AP022110
*Aeromonas* sp. FDAARGOS 1407	78	92.88	4,694,595	CP077399
*Aeromonas phage phiO18P	74	95.33	33,985	NC_009542
*Aeromonas dhakensis* BC03	81	86.2	4,791,622	CP102325
*Aeromonas media* TR3_1	66	86.49	4,521,851	CP075564
*Aeromonas dhakensis* Aer_On24M	75	86.11	4,932,886	CP046626
*Aeromonas salmonicida* 29	84	86.11	4,634,397	CP124840
*Aeromonas caviae* 71,442	66	98.7	4,444,683	CP084350
*Aeromonas dhakensis* 1706–28,330	78	85.98	4,933,619	CP054854
*Aeromonas veronii* AV066	77	85.89	4,702,214	CP126578
*Aeromonas hydrophila* 4,960	57	85.75	4,827,247	CP053883
*Aeromonas phage P05B	81	85.19	32,302	OQ680521
*Aeromonas jandaei* 3,036	75	84.66	4,592,550	CP053882
*Aeromonas hydrophila* AHNIH1	58	86.01	4,906,118	CP016380
*Aeromonas jandaei* 4,608	59	84.48	4,507,629	CP053881
*Aeromonas hydrophila* PartN-Ahydrophila-RM8376	75	87.61	4,733,720	CP064382
*Aeromonas hydrophila* FDAARGOS_916	75	87.61	4,733,702	CP065651
*Aeromonas hydrophila* WP7-S18-ESBL-06	78	87.59	4,940,097	AP022206
*Aeromonas caviae* FDAARGOS_75	75	88.73	4,551,146	CP062801
*Aeromonas* sp. ASNIH4	72	86.63	5,216,518	CP026217
*Aeromonas salmonicida* FN1	67	86.59	5,099,029	CP101948
*Aeromonas* sp. ASNIH3	60	88.38	4,797,236	CP026222

*The asterisk indicates the hit is a culturable isolated phage, while the others are prophages in the bacterial chromosomes.

### 3.9 Phylogenetic analysis

**Figure 6:**
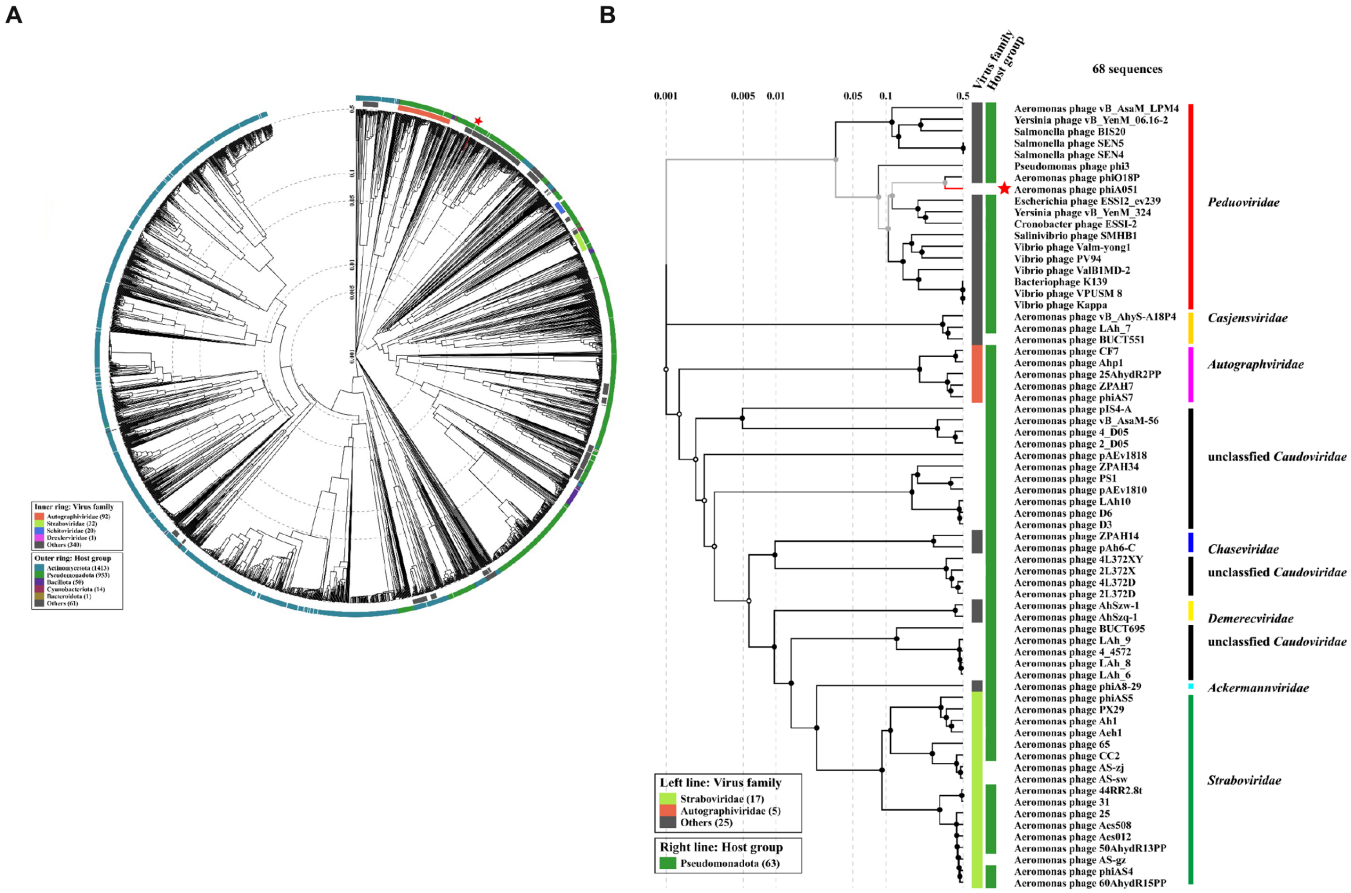
Proteomic trees based on proteomic alignment using VIPTree. **(A)** The viral proteomic circular tree involving phage phiA051 and 2,564 related phages. **(B)** The viral proteomic rectangular tree involving all *Aeromonas* phages and some phages against other hosts. Phage phiA051 is labeled with a red star.

Proteomic trees were constructed by VIPTree (Figure 6). Phage phiA051 was clustered in the family *Peduoviridae* with only two *Aeromonas* phages phiO18P and vB_AsaM_LPM4 (Figure 6B). The largest number of *Aeromonas* phages were clustered in the familys *Straboviridae* and *Autographviridae*.

**Figure 7:**
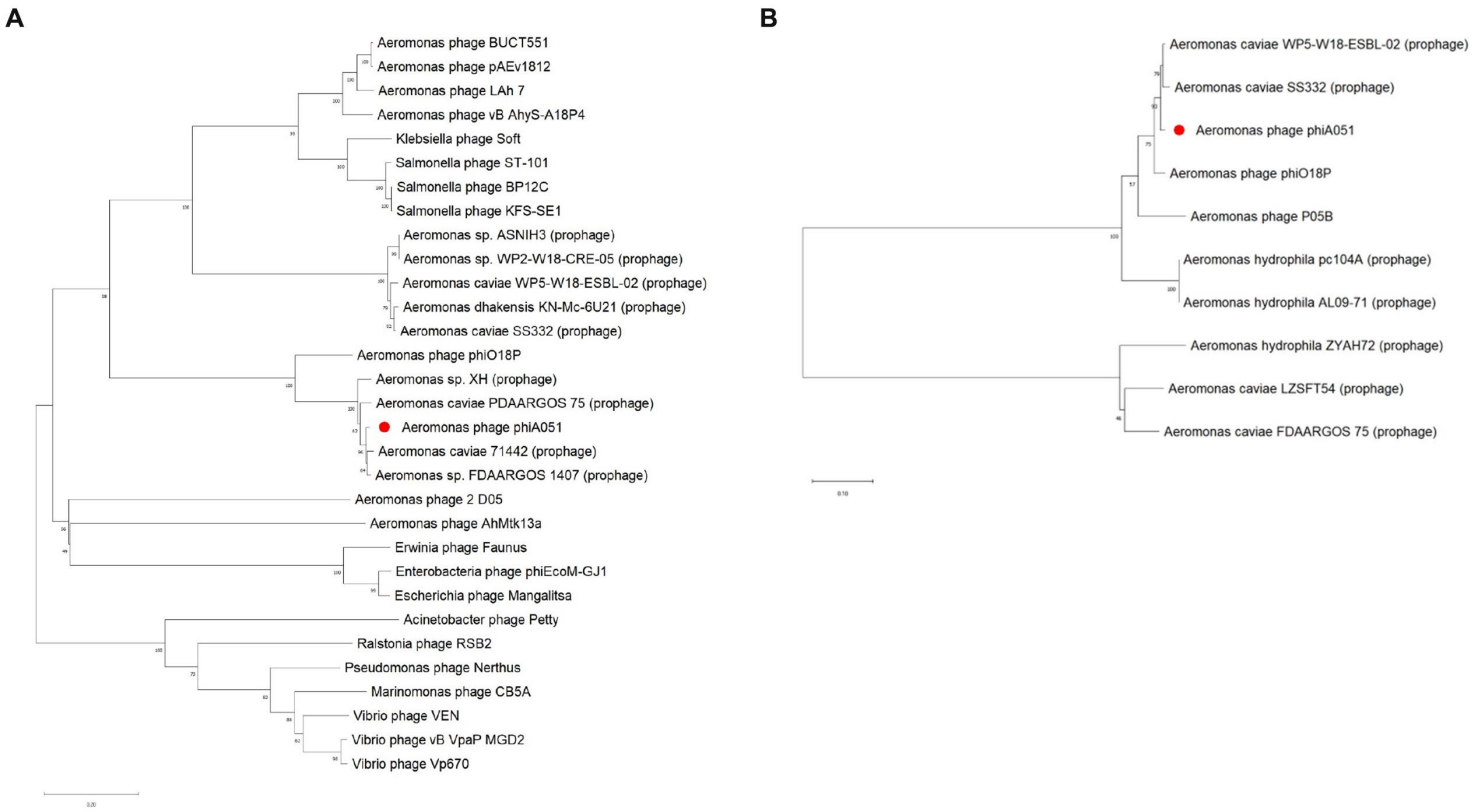
Neighbor-joining phylogenetic tree constructed using the terminase large subunit gene **(A)** and the integrase gene **(B)** with 1,000 bootstrap replicates.

The phylogenetic tree constructed using both the major capsid protein gene and the integrase gene showed that phiA051, phiO18P and SS332 were clustered in the closest branch. However, phylogenetic trees constructed using the terminase large subunit gene show that phiA051 and phiO18P remained on the adjacent branch, while SS332 was on the more distant branch (Figure 7).

### 3.10 Taxonomy

According to the latest demarcation criteria of the International Committee on Classification of Viruses (ICTV) (60), 70 and 95% nucleotide identity of the full genome length were established as the cut-off for genera and species, respectively. The query result by BlastN on the NCBI database showed that phiO18P was the closest isolated phage with phiA051, with an overall similarity of 70.5%. Phage phiO18P had been classified as *Heunggongvirae, Uroviricota, Caudoviricetes, Peduoviridae, Bielevirus, Bielevirus phiO18P* by ICTV, therefore phage phiA051 should be regarded as a novel species in the same genus *Bielevirus* as phiO18P.

## 4 Discussion

In this study, a novel virulent *Aeromonas hydrophila* phage phiA051 was discovered and identified. Although it was only able to lyse 4 out of 24 tested strains of *A. hydrophila*, phage phiA051 could notably inhibit the proliferation of the host bacterium and reduce the final concentration of it, suggesting that this phage has certain potential in rapidly inhibiting the spread of pathogen early in the outbreak. This result likewise suggests that higher phage titers result in better bacterial inhibition, and that the optimal MOI should not be the only reference during practical application. Consistent with previous research findings on bacteriophages, phage phiA051 was found to be not acid tolerant (31, 32, 61–66). The genome of phiA051 comprises 46 open reading frames, of which only 26 are confirmed to be functional. Analyses of the evolutionary position of this phage have shown that it may have originated from a P2-type temperate phage and nonproliferative homologous fragments of this phage exist in several *A. hydrophila* strains, as phiO18P is also a temperate phage and has a ORF of phage major capsid protein of the P2 family in its genome (67). Integrase drives recombination between the phage and specific attachment sites on the host chromosome known as attP and attB during the integration process (68), while holin can accumulate in the bacterial cytosol and eventually penetrate the peptidoglycan layer, and can achieve control of the lytic cycle through self-regulation of its concentration (69–71). This result suggests that the genome of phiA051 is characterized by both temperate phage and virulent phage.

BLASTN comparisons of phage phiA051 showed that the genome of this phage has high similarity to a variety of prophage, the highest of which is *Aeromonas caviae* SS332. It is noteworthy that most of the prophage fragments with high total similarity to phiA051 are close in length and structurally very similar to phiA051, so the result suggests that phiA051 most probably originated from some prophages. In contrast to this possible origin of phiA051, there are not many known proteins associated with lysogeny within the genome of this phage, the most notable of which are CII family proteins. CI and CII-like protein are used to determine the timing of lysis cycle onset and to regulate phage polarity. In particularly, CII proteins are thought to play a key initiating role in inhibiting lytic gene expression and the regulation of the lysogenic cycle of temperate phages (72–74). Despite the annotation of CII family proteins within phiA051, the phage was considered to have lost its lysogenicity in this study considering that the phage exhibited stable lytic ability in culture, but the specific molecular mechanism of the lysogenic shift remains to be thoroughly investigated.

The results of the co-linearity analysis show that the genome of phage phiA051 is significantly different in the location of the gene synteny compared to other phages and prophage that are more homologous to it. It has been shown that the lytic cycle can be restarted by DNA recombination and that may be the opportunity to trigger the process of a temperate phage becoming a virulent phage (75). Myron Levine performed lysogenicity assays on a mutant strain of *Salmonella* temperate phage and demonstrated the possibility of controlling the emergence of lysogenicity by altering the sequence of genes upstream and downstream of the prophage (76), thus regulation of the bacterial genome may impact phage variation. Some studies have since also affirmed the importance of host bacterial genome variation and recombination in the formation of temperate phage mutants (66, 77, 78). Therefore, certain regions in the genome of a strain related to the origin of phiA051 may be similarly involved in the transition between the lytic and lysogenic states. However, in the present study, the upstream and downstream sequences of each prophage with high genomic similarity to phiA051 were examined, and no traces of variation and recombination similar to those reported in the known literature were found, so it was thought that it might be necessary to further explore the origin of phiA051.

Most of the current mainstream research uses induced mutagenesis to isolate possible prophage from known strains to indirectly analyze temperate phages (36, 66, 77–81), and relatively few studies have been conducted on natural mutants, but it was found that phiA051 exhibited the properties of a virulent phage without the need for special treatment, unlike other temperate phages which require stimulation to maintain stable lysis. The discovery of this phage is therefore an important addition to the field. The phage phiA051 was not found potential lysogenic genes such as excisionase and transposase. A number of *Aeromonas* strains that are highly homologous to phiA051 were explored by PHASTER, and it was found that transposase genes were present in some of the strains with prophage. This fact suggests that this gene in genome of phiA051 might have been lost in some case. A study analyzed the gene map of virulent mutants of the temperate phage of *Rhizobium meliloti* and concluded that two mutations in loci avirC and avirT are necessary for the phage to acquire virulent feature (80). However, no similar fragment was found within phiA051. Therefore, further analysis of the lysis mechanism of phiA051 is required.

## 5 Conclusion

In this study, *Aeromonas hydrophila* phage phiA051 was identified by biological and genomic analysis. It was proposed a novel species belong to class *Caudoviricetes*, family *Peduoviridae*, genus *Bielevirus*. The phage showed stable virulent characteristics, but its genome has high similarity to many prophages of *Aeromonas* spp., therefore it is considered to be of probable prophage origin. This discovery has provided a new clue about the prophage in the field of *A. hydrophila* research on temperate phage, and also provided the basis for further research on the origin of viral gene fragments in *A. hydrophila.*

## Data availability statement

The datasets presented in this study can be found in online repositories. The names of the repository/repositories and accession number(s) can be found in the article/supplementary material. The datasets sequence of phage phiA051 for this study can be found in the GenBank under GenBank accession No. OP484843.

## Author contributions

YW: Data curation, Formal analysis, Methodology, Software, Validation, Visualization, Writing – original draft, Writing – review & editing. GT: Data curation, Formal analysis, Methodology, Software, Validation, Visualization, Writing – original draft, Writing – review & editing. XJ: Data curation, Methodology, Writing – review & editing. CT: Data curation, Methodology, Writing – review & editing. HC: Data curation, Methodology, Writing – review & editing. WF: Data curation, Methodology, Writing – review & editing. HT: Data curation, Methodology, Writing – review & editing. QW: Data curation, Methodology, Writing – review & editing. XW: Funding acquisition, Project administration, Supervision, Writing – original draft, Writing – review & editing. ML: Funding acquisition, Project administration, Supervision, Writing – original draft, Writing – review & editing.

## Footnotes

^1^ http://soap.genomics.org.cn/

^2^ http://ccb.jhu.edu/software/glimmer/index.shtml

^3^ http://trna.ucsc.edu/software/

^4^ http://topaz.gatech.edu/GeneMark/

^5^ http://www.ncbi.nlm.nih.gov/BLAST

^6^ https://proksee.ca/

^7^ https://www.genome.jp/viptree/
